# Response to: Rho et al. “Deconstructing Chronic Low Back Pain in the Older Adult-Step by Step Evidence and Expert- Based Recommendations for Evaluation and Treatment. Part VIII: Lateral Hip and Thigh Pain”

**DOI:** 10.1093/pm/pnw296

**Published:** 2017-01-04

**Authors:** Gerard A. Malanga

**Affiliations:** New Jersey Regenerative Institute, Cedar Knolls, New Jersey; Department of PM&R, Rutgers School of Medicine, New Jersey Medical School, Newark, New Jersey, USA

Dear Editor,

It was with great interest that we reviewed the article by Rho et al. “Deconstructing Chronic Low Back pain in the Older Adult-Step by Step Evidence and Expert-Based Recommendations for Evaluation and Treatment. Part VIII: Lateral Hip and Thigh Pain” [1]. We applaud the authors for addressing this common issue of lateral hip pain, and their thoughtfulness and evidence supported discussion regarding the diagnosis of greater trochanteric pain syndrome (GTPS) rather than the commonly used and inaccurate term “greater trochanteric bursitis.” The authors nicely review the literature summarizing that there is a lack of findings on MRI and ultrasound, as well as in histological evidence to support the diagnosis of “bursitis” and/or “tendinitis” in the vast majority of these patients. Additionally, we fully agree with the authors’ noting that there is a lack of inflammatory findings in patients suffering from GTPS and that effective treatment consists of strengthening the weak hip abductors that are often noted in these patients. We have found this to be true in the patients we have treated with GTPS. The authors also note the deficiency of evidence for the use of corticosteroid injections and both the potential systemic side effects and the now well-known toxic effects of corticosteroids to tenocytes, which they note “can potentially contribute to progressive tendinopathy and partial tears.”

It is therefore puzzling that the treatment provided in the clinical case presented in this review was an “ultrasound-guided greater trochanteric bursa corticosteroid injection.” In fact, in this case (as often occurs in clinical practices across the United States) the patient unfortunately underwent two ultrasound-guided corticosteroid injections! This treatment would not only appear to be not indicated based upon the current literature cited in the review by Rho et al., but also potentially harmful in this 90-year-old patient with a history of several compression fractures. In addition, the known lack of efficacy of these injections results in not only a poor outcome but also increased cost. The review failed to include other types of injection treatments for this treatment, including simple needle of the tendon, that is, needle tenotomy, ultrasound-guided dextrose injection, or platelet-rich plasma injection (PRP). In a multicenter case series, Mautner et al. noted a significant benefit using PRP on a variety of tendons, including the hip abductor tendons, namely the gluteus minimus and medius tendons, which commonly demonstrated tendinopathic changes in these patients. Although the current scientific evidence for efficacy of PRP injection is limited, it is superior to the level of evidence for many current treatments for GTPS and certainly superior to corticosteroid injections [2–4]. In our clinical experience, we have found that a single PRP injection of a high-concentration, leukocyte-poor PRP is effective in 90% of patients suffering from GTPS who have failed other conservative measures without the need for additional interventions. In addition, there have been no harmful effects documented in the literature regarding PRP injection of the tendons inserting onto the greater trochanter.

The practice of medicine requires us to provide the most effective evidence-based treatments to patients with the first caveat of “First DO No Harm.” We would argue that injection of corticosteroids for GTPS would not be supported by evidence-based medicine both in terms of effective treatment and avoiding the potential harmful effects.
